# Breed differences in olfactory performance of dogs

**DOI:** 10.1038/s41598-025-87136-y

**Published:** 2025-01-21

**Authors:** Attila Salamon, Ádám Miklósi, László Róbert Zsiros, Tímea Kovács, Enikő Kubinyi, Attila Andics, Márta Gácsi

**Affiliations:** 1 HUN-REN-ELTE Comparative Ethology Research Group, Budapest, Hungary; 2 NAP Canine Brain Research Group, ELTE Eötvös Loránd University, Budapest, Hungary; 3 Department of Ethology, ELTE Eötvös Loránd University, Budapest, Hungary; 4 Hungarian Ethology Foundation, Göd, Hungary; 5 Doctoral School of Biology, ELTE Eötvös Loránd University, Budapest, Hungary; 6 MTA-ELTE Lendület “Momentum” Companion Animal Research Group, Budapest, Hungary; 7 Neuroethology of Communication Lab, Department of Ethology, Eötvös Loránd University, Budapest, Hungary

**Keywords:** Olfaction, Dog breeds, Dog personality, Natural detection Task, Bayesian statistics, Zoology, Animal behaviour

## Abstract

Most studies on dogs’ olfactory performance focused only on some individuals pre-trained for a task or on specially selected/trained detection dogs. Here, using the Natural Detection Task (NDT) that does not require training, we assessed the effect of several potential factors on the performance of a large sample of dogs (*N* = 527/tested, 484/analysed). Olfactory success was associated with breeds, but breed groups (selected for olfaction, cooperation, or both) were proven non-relevant, suggesting that breed-specific traits are more influential than functional breed group characteristics. Border collies, selected for herding, reached higher success levels than golden retrievers, Hungarian/German vizslas and basset/bloodhounds, selected for hunting/olfaction tasks. Beagles found the hidden food quicker than border collies, basset/bloodhounds, golden retrievers, Labradors, and cocker spaniels. Dogs with higher Responsiveness to training personality scores were more successful, while higher training level linked to slower successful search. Activity/Excitability scores, attention-deficit/hyperactivity disorder scores, and owners’ rewarding style were not associated with performance. Overall, large within-breed/breed-group variabilities reflect complex interactions between genetic and environmental factors influencing performance. Given the complex nature of life-like detection tasks, the NDT may provide more information about dogs’ olfactory ability than on their potential effectiveness in detection work.

**Supplementary Information:**

The online version contains supplementary material available at 10.1038/s41598-025-87136-y.

## Introduction

Dogs are our unique partners in detection tasks, in which to be successful, a good sense of smell and the eagerness to cooperate with humans is necessary. Specially trained dogs are used in a wide variety of scent detection tasks in the military and police forces (explosive detection^1,2^; drug detection^3^), in the medical field (disease detection^4–6^; detection of physiological changes in humans^7^), in trailing humans for search and rescue^8^, in human remains detection^2^ and in tracking animals for hunting and conservation^9–11^. Recently, even family dogs of various breeds are extensively used for dog sports relying on their olfactory abilities, such as nose work, mantrailing, tracking, search and rescue (e.g^12^). Studies examining the performance of dogs in olfactory tasks not requiring training are rare (e.g., food search^13,14^).

Although it is widely accepted that dogs have a good sense of smell, it is not quite clear to what extent this ability and their tendency to cooperate contribute to the successful performance of the above tasks. In the study of Polgár et al.^13^, the olfactory performance of breeds selected for olfaction (e.g., hunting breeds) has proven to be better than that of breeds not selected for olfaction. Of note, their results could be partly due to the presence of some breeds selected for cooperation with humans in the olfactory group and their absence in the non-olfactory group, meaning that the olfactory group’s better performance could have been enhanced either by the selection for cooperation alone or by selection for both olfaction and cooperation. Further, it is unknown whether other traits, such as trainability, enhance this performance or if dogs selected for olfaction inherently possess superior olfactory abilities.

Certain breeds have been bred to excel in olfaction-based tasks, so it is reasonable to expect a genetic component to their olfactory ability. However, no breed has been bred exclusively for detection purposes^15^. In drug detection, German shepherd dogs were more successful than Labradors and terriers^3^, but in a study using similar methods Labradors reached the highest proportion of correct indications, while Belgian Malinois were the second and German shepherd dogs the third^16^. In a food search task, Jack Russell terriers, Siberian huskies and French bulldogs performed best, while golden retrievers, miniature Australian shepherd dogs and bichon Bolognese performed worse^14^.

Research into the genetic background of olfactory performance does not show clear results. Even though no differences have been found in the number of olfactory receptor genes between scent hounds, sight hounds, and toy breeds^17^, and between scent breeds and non-scent breeds, as well as between 10 breed-groups defined by the American Kennel Club^18^, differences do exist in the polymorphism of those olfactory receptor genes^19,20^. Further, there is some evidence that genetic differences exist in olfaction related factors^21,22^ and manifest between individuals in the phenotypic level in better scent detection skills, through increased odour recognition efficiency^21^.

Detection dogs have several particular attributes^23,24^ that make certain breeds more favoured. German shepherd dogs, Belgian shepherd dogs and Labradors are commonly used for detection work (e.g^3,25,26^), due to their high stamina and/or high trainability^15,27^. Willingness to engage in persistent olfactory-based searching independently, without the guidance or encouragement of the handler, was considered as an important trait for detection dogs^23,28^.

Since shepherd dogs have not been selected for olfaction, dog trainers and handlers may value their cooperativity more compared to their olfactory ability in detection work. Importantly, so far, there have been no systematic comparative studies in this respect; military and police forces chose detection dogs based on the trainers’ expertise or beliefs. Shepherd dogs belong to breeds selected for visual cooperation with humans. Cooperative breeds were reported to be more successful in a pointing task^29^ and to establish eye contact quicker with the experimenter than non-cooperative breeds^30^, but there was no difference between cooperative vs. non-cooperative working breed groups in their attachment towards the owner^31^.

In the case of olfactory tasks, willingness to work is particularly important^32^. The ability to stay engaged in a task is likely influenced by the desire to obtain the reward for completing the task and how reinforcing the task is. Maejima et al.^25^ showed that ‘desire to work’ – consisting of several underlying traits, such as general activity, ability to obey commands and focus during training – affected the success of drug detection dogs. In detection dogs, high activity level resulting in strong motivation or willingness to work, and high trainability involving attention and low fearfulness probably related to impulsivity, is greatly valued^23^. Factor scores in the Dog Personality Questionnaire (DPQ)^33^ were associated with learning; higher Activity/Excitability and Responsiveness to training was related to more known commands and higher level of basic obedience^34^. Thus, dogs with higher Responsiveness to training and Activity/Excitability scores should perform better.

Dogs’ performance in a task situation may be affected by the behavioural tendencies linked to attention-deficit/hyperactivity disorder (ADHD)^35^. ADHD factors (inattention, hyperactivity and impulsivity) possibly negatively affect performance both in everyday life and in a task situation, reducing the dogs’ ability to concentrate and maintain focus on a prolonged task, and make informed decisions^35–39^.

Moreover, training level may also positively affect dogs’ performance in any test situation (e.g^40,41^), as performing in an unfamiliar environment may be easier if the dog has previous experience with similar task situations or objects used in the test.

In this study, we compared the olfactory performance of family dogs and some professional detection dogs from many breeds in the Natural Detection Task (NDT), developed by Polgár et al.^13^. The NDT is a relatively simple standard method to test dogs’ olfactory ability. It relies on the animal’s inherent motivation to find the target (that is in itself the reward) and it does not require training, in contrast to detection dog studies (e.g^42–44^).

For the present study, breeds were categorised into breed groups based on selection for (1) olfaction but not for cooperation, (2) cooperation but not for olfaction, and (3) both olfaction and cooperation. First, we examined whether breed groups had more homogeneous variance compared to the total variance, which shows whether this categorisation is indeed relevant for olfactory performance in the NDT. In case the categories proved to be relevant, we wanted to compare their performance. Considering that both olfactory abilities and cooperativity may enhance success in the NDT, we did not expect a difference between the two breed groups, however, better performance could be expected from the group selected for both features.

We expected differences between breeds based on previous studies (e.g^3,14,16^). The formulation of more specific hypotheses regarding the breeds was hindered by the huge methodological variation in previous olfactory studies (e.g., applied training, the target scent and the test situation).

Regarding the other examined factors, we expected better performance in the case of: dogs with higher Activity/Excitability and with higher Responsiveness to training scores in the DPQ; dogs with lower ADHD total score; and dogs with higher training level.

## Methods

### Ethics statement

The ethical approval for this study was granted by the Animal Welfare Committee of Eötvös Loránd University (ELTE-AWC-020/2018 and ELTE-AWC-015/2023). All methods were carried out in accordance with relevant guidelines (including ARRIVE) and regulations. The experiment was performed in accordance with the EU Directive 2010/63/EU and the recommendations of the Hungarian State Health and Medical Service. Companion dog owners were recruited through social media and from the Family Dog Project database. The Hungarian Defense Forces 1st „Honvéd” Explosive Ordnance Disposal and Warship Regiment as well as the Rapid Response and Special Police Service, Hungary were contacted directly to recruit professional explosive detection dog trainers and handlers.

Before the test, the owners were asked to fill in a questionnaire, which was carried out in accordance with the relevant guidelines and regulations, including the Declaration of Helsinki, and was approved by the United Ethical Review Committee for Research in Psychology in Hungary (EPKEB; 2023-04). An informed consent was also obtained from all owners and they participated in the test with their dogs on a voluntary basis. Owners could terminate the experiment at any time.

### Subjects

In this study, we tested the olfactory performance of 551 dogs (including 524 family dogs and 27 explosive detection dogs) (> 6 months old) in one of two locations; outdoors or indoors. From these tests, 24 were dropped out due to technical issues and experimenter or owner mistakes, so we could analyse the results of 527 dogs. From these, 43 dogs (including 8 detection dogs) were excluded because they did not pass Level 1, which served as a positive control (8 German shepherd dogs, 7 Belgian Malinois, 4 Basset hounds, 4 Labrador retrievers, 4 golden retrievers, 3 Hungarian short-haired vizslas, 3 border collies, 2 German wirehaired pointers, 2 English bloodhounds, 2 cocker spaniels, 1 beagle, 1 Irish soft coated wheaten terrier, 2 mongrels). Apparently, these dogs either did not understand the task situation or were not motivated enough to search for the food (even though they passed the motivation test, which is explained below).

To assess whether selection for olfaction and/or cooperation in the case of the tested working dog breeds affected performance in the NDT, we created 3 breed groups: breeds that considering their original tasks had been selected for (1) olfaction related tasks, like scenthounds^13^ (Olfactory), (2) cooperation, like sheepdogs^29,31^ (Cooperative), or (3) both olfaction and cooperation, like gundogs (Olfactory and Cooperative). Thus, we evaluated the data of 484 dogs (including 19 detection dogs), comprising 3 breed groups (Olfactory breed group contained 15 breeds, Cooperative breed group contained 6 breeds, Olfactory and Cooperative breed group contained 9 breeds; see Supplementary Table S1). This dataset included 234 males (121 neutered) and 250 females (169 neutered), with ages spanning from 0.5 to 16 years (mean: 4.5, SD: 3.6 years).

To assess the effect of breed, we analysed data from breeds with at least 25 individuals. For this analysis, the term ‘breed’ was used rather loosely, as some closely related variants were merged to maximise the number of dogs in a breed (see Supplementary Table S2). After excluding some dogs due to not fitting into any of our ‘breeds’ (*N* = 13) and to missing questionnaire data (*N* = 32), the performance of 439 dogs (including 10 detection dogs) encompassing 10 ‘breeds’ were compared. This set contained 211 males (116 neutered) and 228 females (157 neutered), with ages ranging from 0.5 to 16 years (mean: 4.6, SD: 3.7 years). We tried to balance the breeds for age and test location as much as possible.

### Location

**Fig. 1:**
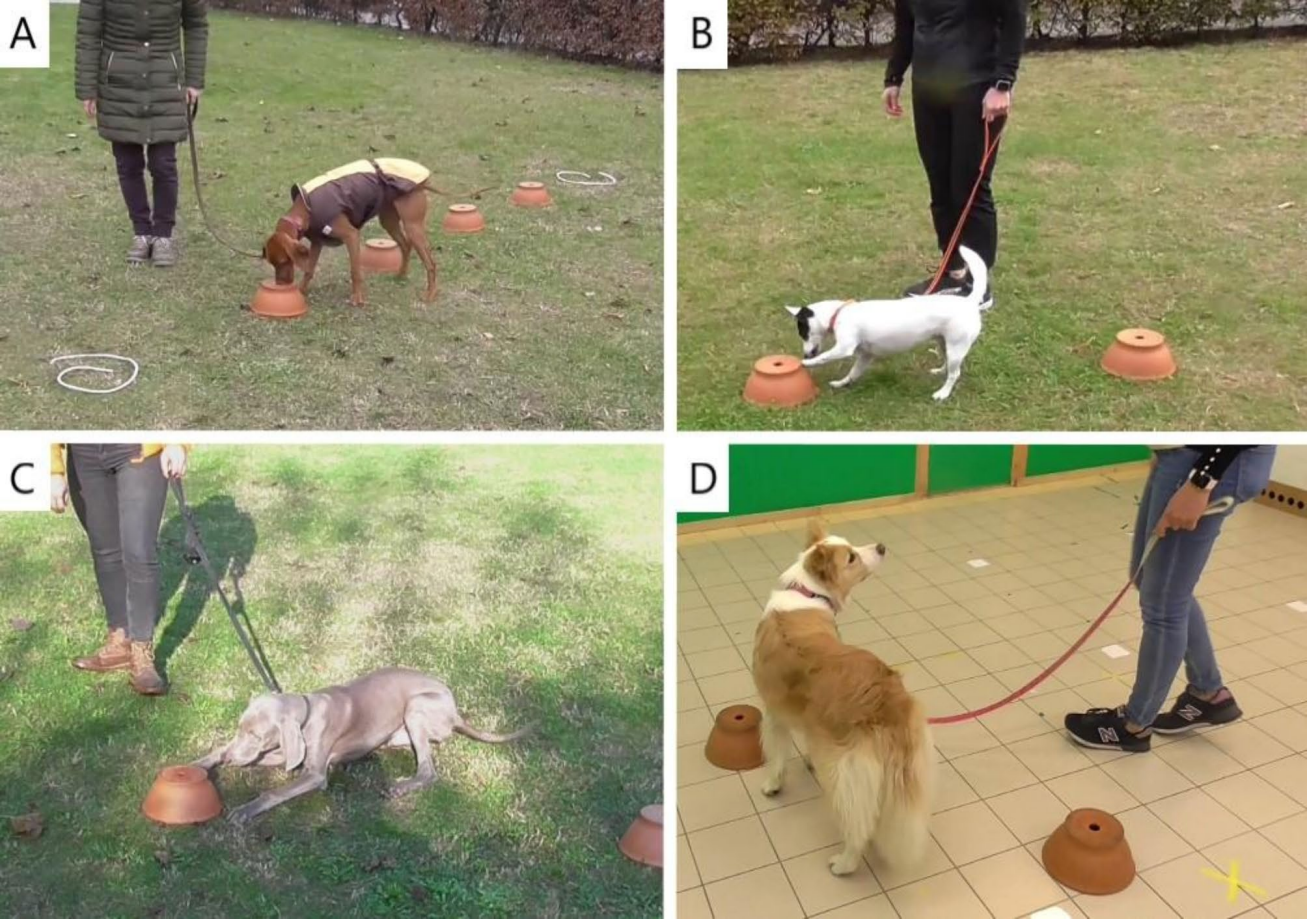
Test locations and indications. A–C: Outdoor location; flat grassy area surrounded by hedges and trees, relatively isolated from pedestrians and dog walkers. D: Indoor location; clean and empty laboratory room. Dogs’ spontaneous indications of the target scent (4 typical types): A – nose in the hole for > 2 s, B – pawing, C – lying down, D – looking at the owner.

The tests were conducted both outdoors and indoors, depending on the weather and lab availability. The outdoor area (see Fig. 1A–C) was a flat grassy area surrounded by hedges, thus relatively visually isolated from potential distractions. This area was not frequented by dog walkers, minimising the amount of distracting scents or visual effects. The indoor location was a 6.3 × 5.4 m test room at the Department of Ethology, Eötvös Loránd University (see Fig. 1D) that was continuously ventilated and thoroughly cleaned with cleaning agents, which did not contain perfume, after each test.

The active explosive detection dogs were tested in a familiar clean and empty room at their service location, either at the Hungarian Defense Forces 1st „Honvéd” Explosive Ordnance Disposal and Warship Regiment or at the Rapid Response and Special Police Service.

### Procedure

The testing procedure was based on Salamon et al.^45^, which is an adapted form of the NDT (see^13^). This testing procedure differs from a traditional scent test for trained dogs (e.g., line-up^46,47^), as the dogs in the NDT were not trained before or during the test; instead, their natural motivation for the baited food is utilised. Following the recommendation of Polgár et al.^13^, we implemented three levels instead of the original five, alongside modifications in the evaluation methods (detailed below).

Before starting the test, the dog’s motivation was evaluated by offering a piece of bait from an open container placed on the ground in front of the dog. The test would only begin if the dog ate the bait from the container. For all dogs, the hypoallergenic Alpha Spirit Only Fish semi-moist food was used as bait.

Throughout the test, four round ceramic pots (23 cm in diameter), each with a 2.5 cm hole at the bottom, were arranged upside-down in a linear fashion, spaced at approximately one-metre intervals. Starting lines were placed 1.5 m away from each end of the pot line.

Underneath each pot, a 0.25-L plastic container with a lid (Curver brand) was placed. There were dedicated ‘Food’ and ‘No food’ containers/lids to prevent the confounding effects of residual odours. In each trial, from the four containers one was a ‘Food’ container/lid that contained the bait, and three of the containers were ‘No food’ containers/lids that never came into contact with any food or any persons who handled the food. Several additional containers were used for baiting, allowing for regular replacements.

Two experimenters (E1 and E2) conducted the test. E1 arranged the pots and placed the baited container in the predetermined position using a teaspoon for bait placement without direct hand contact. The baiting was done in a pre-fixed pseudorandom order, that is, the bait was hidden under each pot once in every four trials and it was never under the same pot twice consecutively. E2 never came into contact with the bait, the pots or the containers, and was unaware of the baited container’s location. E2 explained the test procedure to the owner and recorded the trials using a handheld camera (Panasonic HC-V180) or a smartphone. The owner and the dog did not view the baiting of the pots and faced away from the test area until they were called. In the case of indoor tests, the owner and the dog left the test room for the time of baiting^45^.

Once the bait was placed under a pot, E2 started a stopwatch. When E2 placed the food in the container under the pot, its scent began accumulating (first in the container, then in the pot). We waited one minute following the methods of Polgár et al.^13^ and Salamon et al.^45^ to ascertain the time necessary for the odour to flow out to a sufficient extent. After one minute had elapsed, E1 called the owner and the dog to come to the starting line, allowing them to begin walking the path on a loose leash. Before they started walking, the owner was allowed to give an encouraging command (such as “search!”, “sniff!”, or “go find!”), but no talking or gestures were allowed while walking or when the dog examined the pots. The only exception was calling the dog back with an encouraging tone, if it moved away from the setup, saying: “come here, where’s the food?” (see^45^).

While walking along the setup, the dog could sniff each pot. As dogs were not trained for any type of indication, the trial concluded when the dog spontaneously indicated a pot or after one minute of active searching without indication. A spontaneous indication was defined as any of the following behaviours: placing paw on pot; refusing to move away from pot; attempting to turn the pot over; poking the pot with the nose; sitting or lying down next to the pot; vocalising while next to pot; significantly increasing tail wagging speed while sniffing pot (see Fig. 1A–D). E2, who was blind to the location of the bait, determined the presence of an indication, which only counted if lasting more than 2 s. If the indication lasted less than 2 s and the dog moved on, it was allowed to keep searching. If the dog turned the pot over, it was an indication, and the owner had to grab the container at once without waiting for E2. If the owner and the animal reached the end of the line without indication before the minute elapsed, they could turn back and walk the line in the opposite direction until the time was up. When turning back, the owner again was allowed to encourage the dog at the line. During the 1–2-minute waiting period between trials, the dog remained on leash, was allowed to drink and rest (see^45^). The entire test took 35–75 min, depending on the dog’s performance.

The outcome of each trial fell into one of four categories (see^13,45^): correct choice, incorrect choice, no choice, and no attempt.

Correct choice: The choice was considered correct if the subject indicated the baited pot within the allotted time. The owner lifted the pot, opened the container, and let the dog eat the bait. Praising was allowed, but the owner was not permitted to touch the bait by hand.

Incorrect choice: The choice was incorrect if the subject indicated an unbaited pot. In that case, the owner removed the lid and showed the dog that the container under the indicated pot contained no food.

No choice: No choice was recorded if the subject examined all four pots without making an indication within one minute of active searching.

No attempt: No attempt was recorded if the subject did not examine all the pots, showed no interest, or repeatedly moved away from the pot line. In case of no choice or no attempt, none of the pots were shown to the dog. The test finished after three consecutive no-choices and/or no attempts.

E1 replaced the baited container, the lid and the pot with clean ones for the next trial. They were ventilated for one trial and then reused as a non-baited container (the incorrectly indicated pot and container were not replaced).

Based on experiences from the pilot studies and aiming to facilitate rapid learning of the task, we adopted a method employing three progressively challenging levels, as suggested by Polgár et al.^13^. The difficulty levels were categorised based on the type of lid placed on the baited container (see^45^).

In Level 1, the containers had an open top with no lid. Serving as a pre-test, this level is primarily controlled for (a) understanding the setup/task, (b) motivation in a task situation (to act independently), and (c) the utilisation of alternative problem-solving methods aside from olfaction (such as turning all pots upside down). Data from dogs failing this level were not subjected to analysis.

In Level 2, the containers were covered with lids that had five holes, each 0.5 cm in diameter.

In Level 3, the containers were covered completely with a regular lid (no holes). Even though one would expect that the odour would not evaporate from the sealed container, previous studies suggest that such containers are not airtight^13,45^.

The subject advanced to the next level, if it made at least 3 correct choices from the last 4 trials. If a subject could not advance within 12 trials to the next level, the test concluded. This meant a minimum of 3 and a maximum of 12 trials at each level, totalling 3–36 trials (see^45^).

In order to minimise the likelihood of the subjects’ use of cognitive strategies or local enhancement to indicate a pot^48^, the row of pots was rotated to a different angle and shifted over at least 1 m after each trial by E1 on the field or in the test room. Additionally, the starting line was switched to the opposite side for each trial.

### Questionnaires

We used the Dog Personality Questionnaire (DPQ)^33^ to assess dog personality. The DPQ consists of 45 items, and the owners were asked to score how much they agreed with each statement using a 7-point Likert scale. The questionnaire assesses five factors consisting of 2 to 4 facets, and each facet is composed of three questionnaire items^33^. The questionnaire item scores were averaged in each facet, and the facet scores were averaged in each factor. The five factors were labelled as follows: Fearfulness, Aggression towards people, Activity/Excitability, Responsiveness to training (henceforth referred to as Responsiveness), Aggression towards animals. From the above factors, we had hypotheses for the Activity/Excitability and Responsiveness factors and used the scores (1–7) as continuous variables.

We used the Dog ADHD and Functionality Rating Scale (DAFRS) to assess ADHD-related traits of the tested dogs^38^. The DAFRS questionnaire consists of 17 items, and the owners were to score how frequently they experienced the behaviours of their dogs mentioned in the questionnaire using a 4-point Likert scale. The questionnaire assessed three factors: inattention (6 items), hyperactivity (4 items) and impulsivity (7 items). The scores of the items were summed to get the factor scores. The sum of the factor scores yielded the ADHD total score. It is important to note that none of the dogs were diagnosed with ADHD.

Along with the DPQ and DAFRS questionnaires, we also collected data on the dogs’ training level and how the dogs were typically rewarded in task situations. The training level was categorised as follows: 0 – no training, 1 – basic obedience, 2 – special training (e.g., agility, therapy), 3 – special olfactory training (e.g., mantrailing, scent work, hunting, truffle search, and this category included all detection dogs).

The owners’ general rewarding style, that is, if treats play a crucial role in their rewarding regime, can potentially affect dogs’ performance. Thus, we counterbalanced our sample for this factor as much as possible and also included it in the analysis, as in family dogs, high food motivation may be an advantage in the NDT. The owners’ general rewarding style was divided into two categories: dogs that were exclusively rewarded with food in everyday life, and dogs that were rewarded not exclusively by food but also with toys and/or social reward. (Since most owners rewarded their dogs with food, we could not make a distinction between dogs only motivated with food and dogs only motivated with toys and/or social reward).

All tests were carried out in Hungary, but some owners were foreigners, therefore we used the original English versions of the questionnaires in addition to the Hungarian versions. The owners filled in the questionnaires online.

### Data pre-processing

Data pre-processing and cleaning were performed using Python (v. 3.11.3)^49^ with the pandas library (v. 2.0.1)^50^. The behavioural coding was done using the Behavioral Observation Research Interactive Software (BORIS version 8.2)^51^.

We utilised three main metrics to evaluate the olfactory performance of dogs:

Top level (TL): It is a Boolean variable, indicating whether a dog managed to pass the third and most challenging level of the NDT or not.

Success Score (SUS): It is a multifaceted, ordinal metric ranging from 1 to 4 that integrates the levels a dog passed, and the number of trials taken to achieve those levels. The SUS metric was developed in a way to be more sensitive to dogs’ performance compared to TL, but each of its categories contain a large enough sample size for meaningful statistical analysis (see^45^). The specific calculation for the SUS can be found in Supplementary Table S3, while the illustration of these two metrics used to characterise the subjects’ performance in the NDT is shown in Supplementary Figure S1.

Successful Level Latency (SLL): Since dogs were not trained for the NDT, we did not record their false positive and false negative choices, instead we measured latency to compare the speed of their successful choices. To calculate SLL, for each dog we considered the mean latency of the successful trials on their last successful level. We only considered the last successful level, as it was the dogs’ peak performance when completing the task successfully using their olfaction. We only considered the successful trials on the last level, as the unsuccessful trials would not provide information on olfactory success.

Four coders coded a subset of 4% of the videos to assess inter-rater reliability (using Cohen’s Kappa) for the latency variable. A high level of agreement was found between all coders (Coder 1 vs. Coder 2, K = 0.902; Coder 1 vs. Coder 3, K = 0.937; Coder 1 vs. Coder 4, K = 0.972; Coder 2 vs. Coder 3, K = 0.886; Coder 2 vs. Coder 4, K = 0.79; Coder 3 vs. Coder 4, K = 0.928). Four dogs had no latency data due to technical problems.

### Statistical analysis

All statistical analyses were conducted in R (v. 4.3.2)^52^ using RStudio (v. 2022.7.1.554)^53^. Basic statistical analyses were performed using native R statistical functions. The tidyverse suite of packages facilitated data manipulation and transformation as well as visualisation^54^.

First, we examined whether the categorisation by breed groups (based on selection for olfaction and/or cooperation) was a viable option apart from the breed categorisation. Thus, we compared the variance of the breed groups to the overall variance using the F test of homogeneity of variance. This analysis was aimed to determine whether breed groups will be used later in the models.

To investigate the potential effect of the breed, Activity/Excitability, Responsiveness, ADHD total, training level, rewarding style and age on the sample (for all metrics) containing dogs of 10 breeds (*N* = 439), we turned to Bayesian generalised linear regression models (GLM). In the models we included test location as a random factor to control for its effect based on the findings of Salamon et al.^45^. The interaction of breed and training level was also examined, but since it did not improve the models, it was excluded. The choice to employ a Bayesian approach was motivated by our aim to incorporate prior knowledge and achieve a more nuanced understanding of the underlying distributions and effects, especially given the complex nature of our data.

Bayesian analyses were carried out using the brm function from the brms package^55^, specifically employing binomial model for binary outcome TL, cumulative link model for the ordered categorical SUS, and Gamma distribution with logarithmic link function for the positively skewed continuous latency (SLL). Each of the three response variables (TL, SUS and SLL) was modelled to assess the influence of several predictors. These predictors included breed, Activity/Excitability, Responsiveness, ADHD total, training level and rewarding style related scores, as well as age. In each model we used test location as a random variable to account for the variability in potential confounding factors that may be specific to test location (see^45^). Reanalysis with the respective frequentist counterparts yielded similar results, confirming the robustness of our data and supporting the reliability of our Bayesian approach. For features previously unstudied, we utilised uninformed priors (normal distribution centred at 0 with a standard deviation of 2.5) after confirming through a sensitivity analysis that these priors provided a robust fit to our dataset. We used previous data on the grouping variable, test location, from Salamon et al.^45^, applying these posteriors as priors for our current models: TL (normal (0, 0.22)) and SUS (normal (0, 0.19)). In the Monte Carlo Markov Chain process there were 2000 iterations, running four chains in parallel to ensure precise estimation. The sensitivity analysis was conducted using at least three sets of priors of varying widths for each model (standard deviations of 2.5, 1, and 5). This analysis confirmed the robustness of our models, as evidenced by fairly low differences in estimates, at least moderately high bulk effective sample sizes, and Gelman-Rubin convergence statistics (R-hat values) consistently at 1. Based on the sensitivity analysis, we adjusted some priors to refine our models and enhance the reliability of our parameter estimates. All our Bayesian GLMs were selected based on thorough consideration by evaluating commonly used frequentist (e.g., Akaike Information Criterion, adjusted R-squared) and Bayesian performance metrics (e.g., Leave One Out Cross-Validation, Watanabe-Akaike Information Criterion), as well as by visually inspecting graphical diagnostic plots (Posterior Predictive Check). For each of the Bayesian GLMs, we estimated the posterior distributions of the model coefficients (log odds) for the predictors. Odds ratios (ORs) were then calculated by exponentiating these coefficients (i.e., OR = exp(coefficient)). This transformation provides a multiplicative measure of the effect of each predictor on the odds of the outcome occurring.

For better interpretability of our results, it is important to note that the Bayesian statistical framework approaches probability differently from frequentist (Fisherian) statistics. In Bayesian analysis, statistical inference is based on the posterior distributions of the parameters, which result from combining prior information with the observed data^56^. In this paper we summarise the parameters’ posterior distributions using odds ratios and their corresponding 95% credibility intervals (CI). The ORs represent the estimated effect sizes, where an OR greater than 1 indicates increased odds of the outcome per unit increase in the predictor, and an OR less than 1 indicates decreased odds. The 95% CIs are calculated by extracting the 2.5th and 97.5th percentiles from the posterior distributions. These intervals provide the range within which the true parameter values lie with 95% probability, given the data and the model. Therefore, instead of p-values, we focus on these Bayesian estimates to convey the strength and direction of the observed effects (see Supplementary Table S4). We consider a factor having an effect, if the entire 95% of the CI excludes 1 (i.e. credibly different). Please note that we use the term “significant” in this paper for ease of interpretation for readers less accustomed to Bayesian statistics. However, “significant” in this context does by no means carry the same meaning as significance in frequentist statistics.

## Results

### Breed groups

In order to find out whether the categorization of breed groups based on original function (selected for olfaction, cooperation, or both) has relevance in this olfactory task, we examined whether the variance within the breed groups was more homogeneous compared to the variance of the overall sample. For all three response variables (TL, SUS, SLL) the breed group variance did not differ from the overall variance within our dataset (*p* > 0.05 for all breed groups for each response variable) showing that this categorisation is not viable. Therefore, we did not analyse the breed group data, and the breed group variable was not considered in further analyses. (To illustrate the variability within the breed groups, in the figures we grouped the breeds based on the original categories.)

### Breeds

**Fig. 2:**
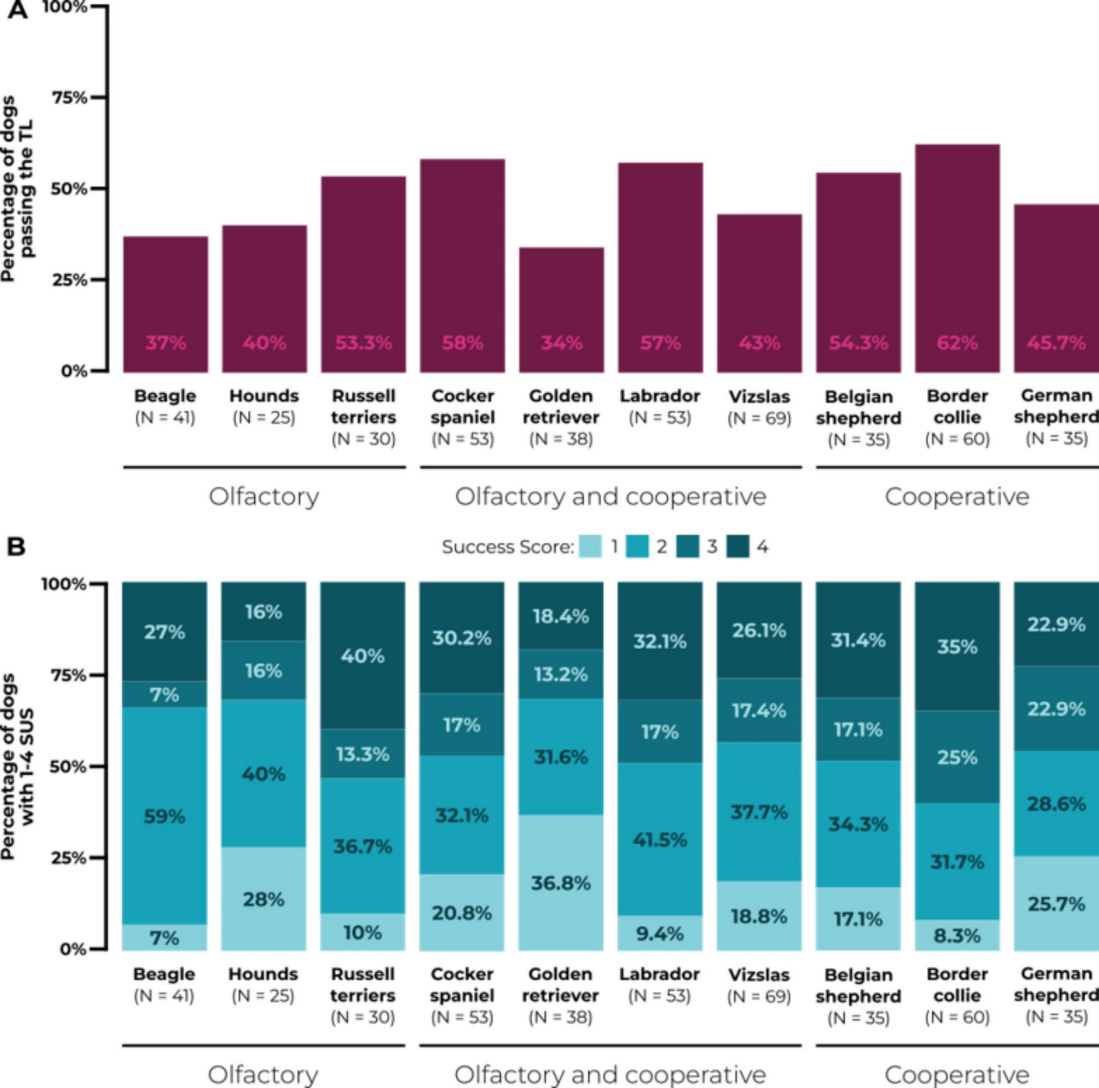
A: The percentage of dogs passing Top Level in each breed (total *N* = 439). B: The Success Score (1–4, where 4 is the most successful) results of each dog breed (total *N* = 439). Hounds include basset and bloodhounds.

**Fig. 3:**
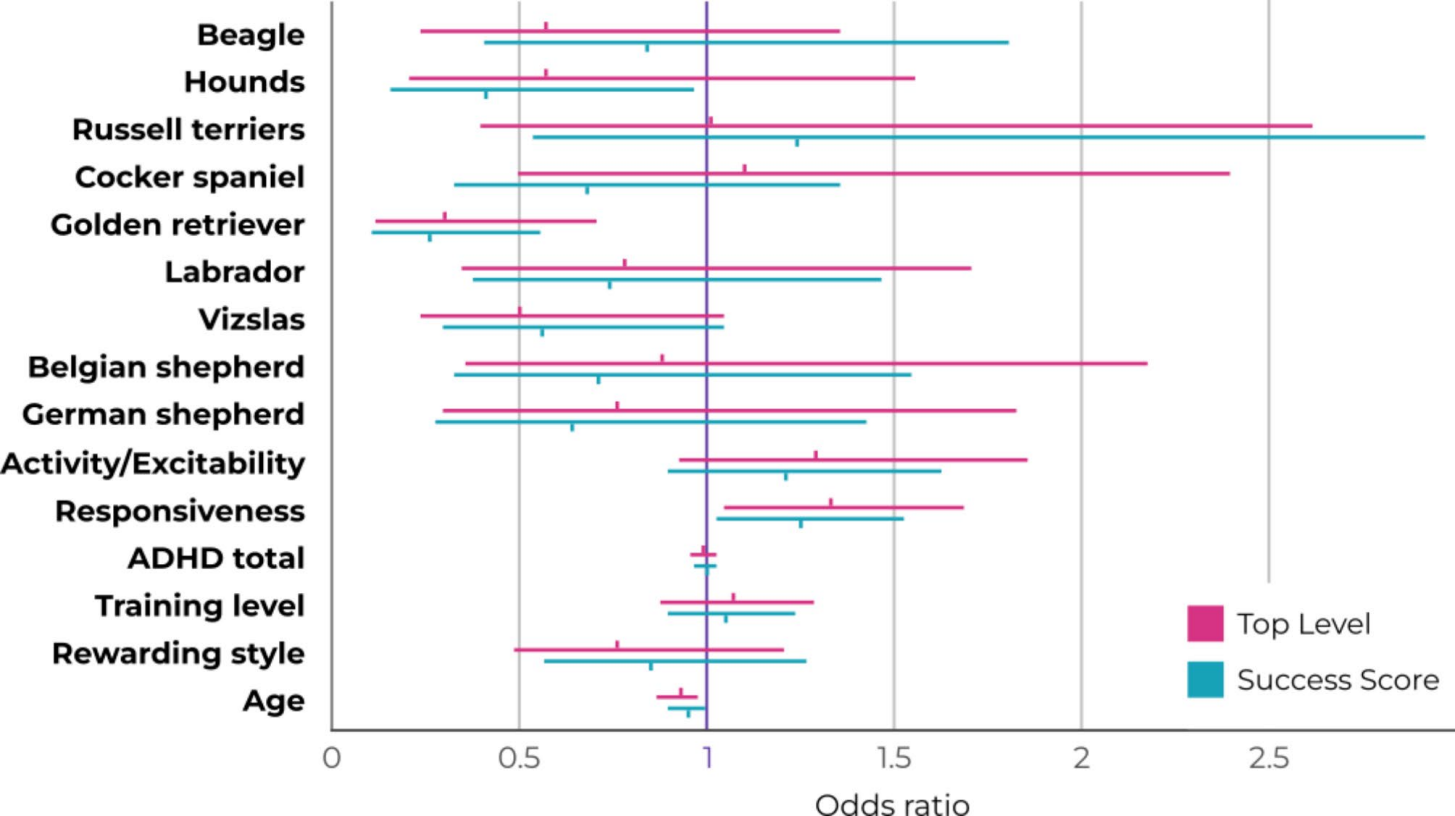
Odds ratios and the lower and upper 95% of their credibility intervals (CI) for the measured variables in relation to Top Level and Success Score. The border collie was the reference breed for both Top Level and Success Score. The effect of a factor can be considered significant if 1 does not fall between the L-95% CI and U-95% CI values. The uncertainty of the effect of a factor increases with the width of the CI range, and the distance of the odds ratio from 1. (Hounds include basset and bloodhounds.)

Border collies passed the TL in the highest percentage (Fig. 2A); thus, we used them as the reference breed for the statistical analysis of the TL and the SUS. Fewer golden retrievers and vizslas passed the TL compared to the reference breed, with decreases in the odds by 70% and 50% respectively, though in case of the latter the U-95% CI did encompass 1, suggesting slight uncertainty in these results (Fig. 3, Supplementary Table S4). In the case of the other breeds, the wide range of CI and the position of 1 within the CI suggest a lack of difference compared to the reference breed.

Golden retrievers, hounds (basset/bloodhounds) and vizslas achieved lower SUS compared to border collies, the reference breed, with decreases in the odds by 74%, 59% and 44% respectively (Figs. 2B and 3, Supplementary Table S4). However, the U-95% CI for vizslas encompassed 1, hinting a slight uncertainty in these results. In the case of the other breeds, the wide range of CI and the position of 1 within the CI suggest a lack of difference compared to the reference breed.

**Fig. 4:**
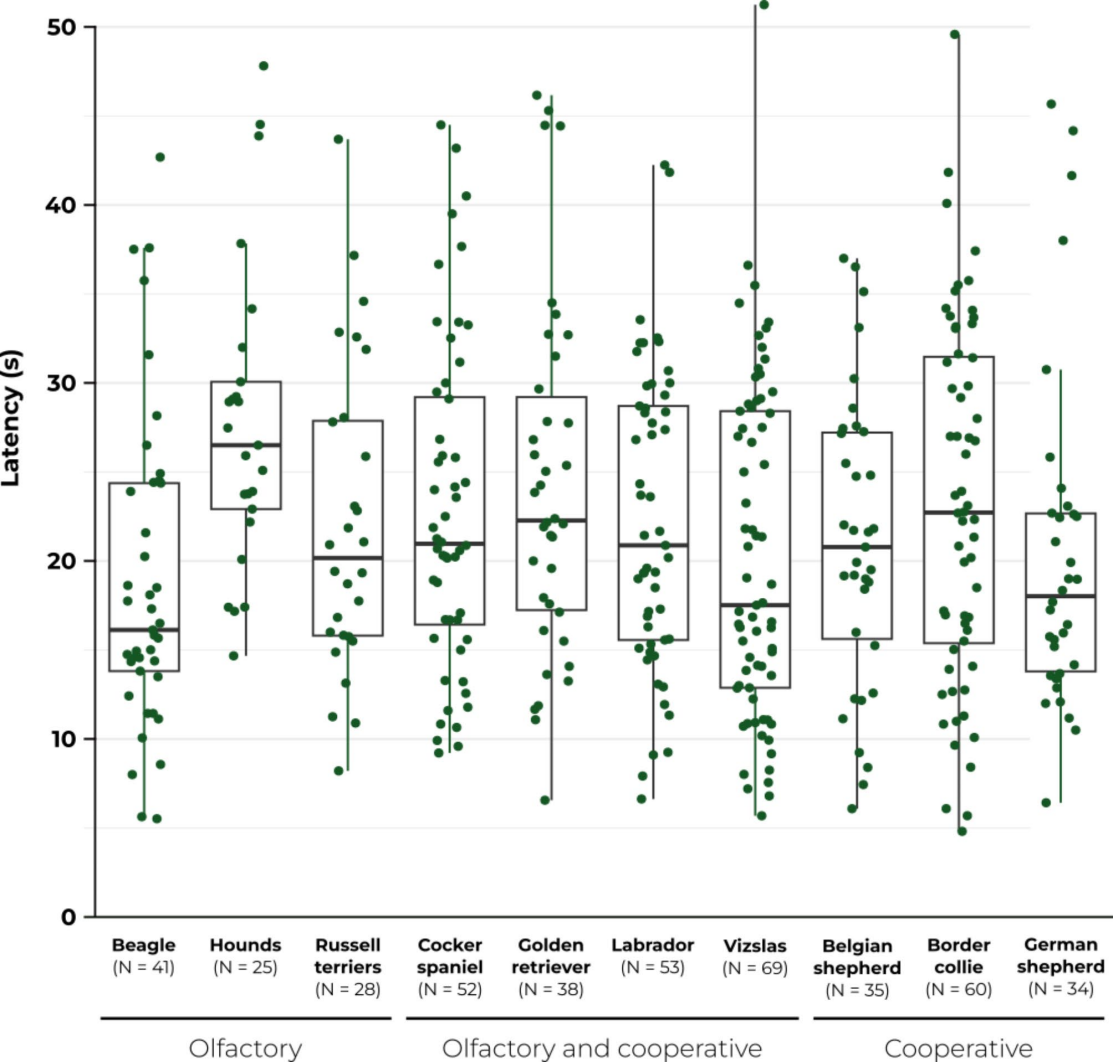
The Successful Level Latency for each dog breed (total *N* = 435). The Successful Level Latency boxplot graph shows median, quartiles, minimum, and maximum with individual data points jittered. Hounds include basset and bloodhounds.

**Fig. 5:**
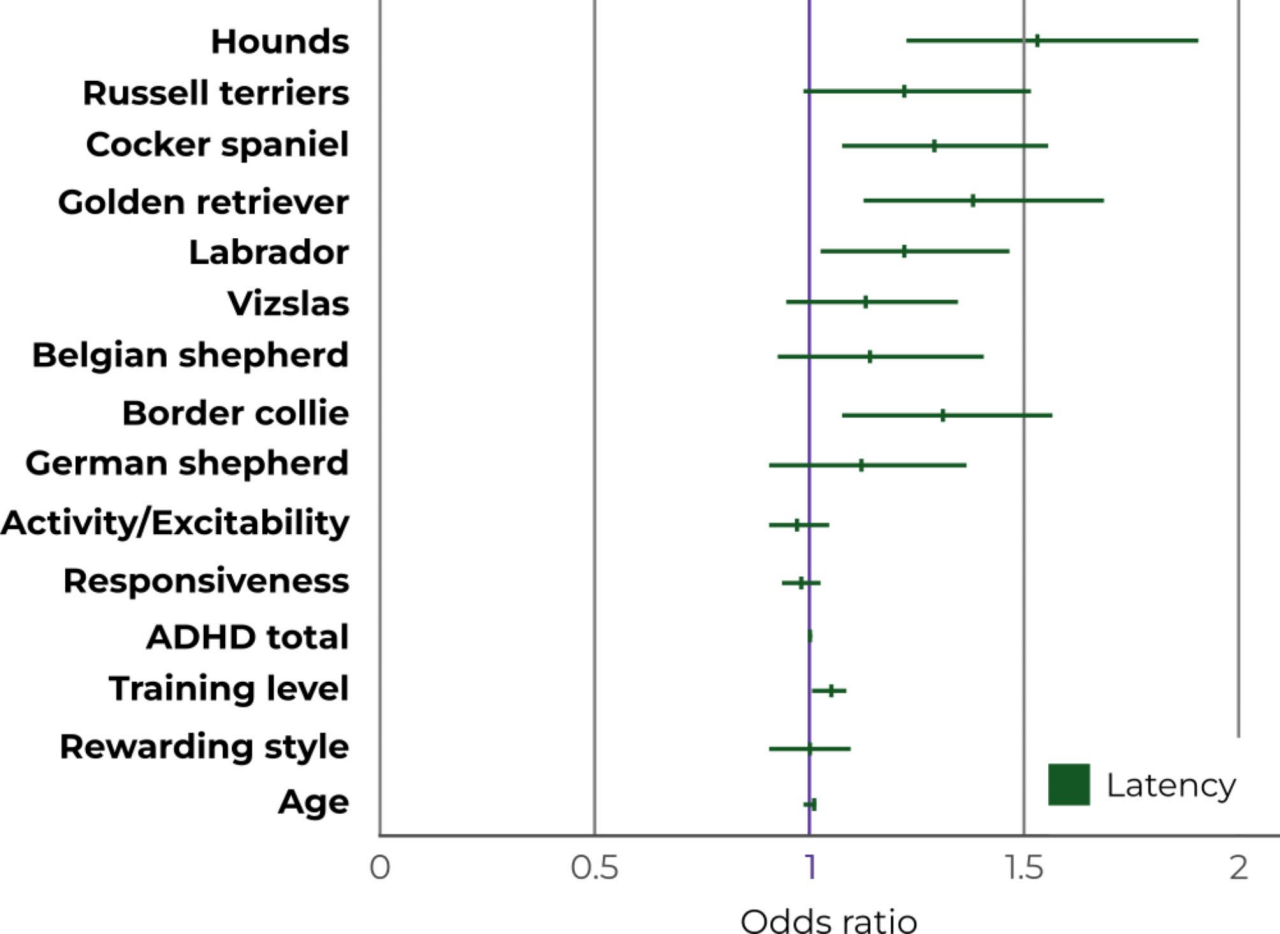
Odds ratios and the lower and upper 95% of their credibility intervals (CI) for the measured variables in relation to Successful Level Latency. The beagle was the reference breed. The effect of a factor can be considered significant if 1 does not fall between the L-95% CI and U-95% CI values. The uncertainty of the effect of a factor increases with the width of the CI range, and the distance of the odds ratio is from 1. (Hounds include basset and bloodhounds.)

Beagles completed their last successful level the quickest (Fig. 4); thus, we used them as the reference breed for the statistical analysis of the SLL. Hounds (basset/bloodhounds), golden retrievers, border collies, cocker spaniels, Labradors and Russell terriers had lower SLL, i.e., on average passed the last successful level slower, compared to beagles (Fig. 4), with increases in the odds by 53%, 38%, 31%, 29%, 22% and 22%, respectively (Fig. 5, Supplementary Table S4). However, the L-95% CI for Russell terriers touch upon 1, hinting a slight uncertainty in these results. In the case of Belgian shepherd dogs, German shepherd dogs and vizslas, the wide range of CI and the position of 1 within the CI suggest a lack of difference compared to the reference breed.

### Personality (DPQ), ADHD total, training level, rewarding style, and age

Dogs with higher Responsiveness scores more likely passed TL and achieved a higher SUS with increases in the odds by 33% and 25%, respectively (Fig. 3, Supplementary Fig. S2, Supplementary Table S4). Older dogs were less likely to pass TL and even less likely to achieve higher SUS with estimated decreases in the odds by 7% and 5%, respectively (Fig. 3, Supplementary Table S4). Since an OR of 1 represents no effect, we interpret these effect sizes credible but modest.

In the case of the other examined factors (Activity/Excitability, ADHD total, training level, rewarding style), the relatively wide range of CI and/or the position of 1 within the CI suggests no effect on the TL and SUS.

Dogs with a higher training level had higher SLL, that is, on average they passed the last successful level slower, with an improvement in the odds by 5% (Fig. 5, Supplementary Table S4). The position of 1 within the CI suggests no effect of Activity/Excitability, Responsiveness, ADHD total, rewarding style and age on the SLL (Supplementary Table S4). The training and breed interaction did not improve the models; therefore, it was excluded.

## Discussion

This study examined the effects of breeds, breed groups (selected for olfaction, cooperation or both), dogs’ personality and ADHD factors, training level and the owners’ rewarding style on the olfactory performance of dogs tested in the NDT. The breed group variance did not differ from the overall variance within our dataset, suggesting that this type of breed group classification is irrelevant in this context. Though in most cases the within breed variability seemed to be also relatively high (see Figs. 2B and 4), the breed affected both success and speed in the olfactory task. From the other examined factors only the training level and the Responsiveness could be linked to olfactory performance in the NDT.

The breed groups we constructed based on breeds’ original selection for olfaction and/or cooperativity, did not prove to be a useful categorisation to examine dogs’ olfactory performance. This complements the findings of Mouton et al.^18^ who examined some olfactory-related genetic and anatomic features of dogs, and neither found difference among functional groups, for example, scent vs. non-scent breeds, nor among genealogical groups defined by the American Kennel Club. The large variability between breeds selected for similar abilities/traits may be partly explained by the relaxed selection in the case of some breeds in recent decades, which diminished the presumably once existing differences between breed groups in olfactory performance. However, this seems to contradict the results of Polgár et al.^13^, who found that the group of scent breeds outperformed the group of non-scent breeds in the NDT. In that study, however, only the scent breed group contained cooperative working dog breeds (gundogs), which could not be ruled out to contribute to their success. Based on our recent results, however, it seems that the combined selection for cooperativeness and olfactory abilities does not result in a more homogeneous group in terms of success. Thus, the summarised results of the two studies suggest that both the selection for olfaction and for cooperation matters in olfactory performance, but their effects do not simply add up. Moreover, based on the large within-group variability in the performance of our three categories, we can assume an effect of relaxed selection also for cooperativity in the case of at least some breeds (e.g., golden retrievers).

In contrast to breed group categories, the studied breeds proved to be different in several aspects of their performance. Due to the wide range of differences in the applied methodology (e.g., various subjects, target scents, test environments) of previous studies that examined the olfactory performance of different breeds, it is quite challenging to compare previous results with those of the present study. Apart from the testing methodology and the large variation in the number of subjects used, the results can be affected by several confounding factors, such as demographic or environmental factors, which are controlled differently or not at all. Since working and show lines exist in several breeds, there can also be differences due to these behavioural phenotypes^57,58^. Further, the sampling (convenience vs. hypothesis-driven) methodology may impact the results and the drawn conclusions of a study comparing breeds^59^. We aimed to at least partly overcome these challenges using an exceptionally large sample size in this study.

In spite of the great within-breed variability, indicating that individual genetic and environmental factors play a crucial role, we found significant breed differences in their success. Compared to border collies, a lower proportion of golden retrievers and vizslas passed the TL, and a lower proportion of golden retrievers, hounds and vizslas reached a higher SUS. Of note, the NDT is most similar to a “line up” of scent boxes/containers that can be recognised visually as targets, which are used in foundational training and for odour recognition tests but are not an integral part of detection dog’s operational work, where dogs need to locate hidden odour in the real environment. Thus, for example, border collies’ remarkable success in the task might be partly related to their highly visual type of work, which may be an advantage due to the visual (choice task) nature of the NDT. The border collie is rarely examined in comparative studies of olfactory performance. While in the UK, springer spaniels and Labradors are favoured breeds for drug and explosive detection and in a survey 16.7% of explosive detection dog trainers and handlers expressed a preference for border collies after these breeds^60^, in Europe most law enforcement units use German shepherd dogs, Belgian Malinois and Labradors for detection work^61,62^. Belgian Malinois were reported to be more successful than German shepherd dogs in an explosive search test (police explosive detection dogs)^63^ and in human tracking (police detection dogs)^62^, but not in drug/tobacco detection (customs detection dogs)^64^ and in the NDT (family dogs) in the present study. Further, in contrast to previous studies with drug detection dogs, we did not find a difference in the olfactory success of German shepherd dogs, Belgian shepherd dogs, cocker spaniels, Labradors and Russell terriers^3,16^. It has to be noted that the above studies all tested professional detection dogs with trained target scents, while we tested family dogs exploiting their natural food motivation in the NDT, which could explain some of the differences between the results. However, Ferrando and Dahl^14^ tested various dog breeds in a food detection task and found that Jack Russell terriers, Siberian huskies and French bulldogs performed best, while golden retrievers, miniature Australian shepherd dogs and bichon Bolognese performed below chance level. Their results on Jack Russell terriers and golden retrievers seem to be consistent with ours, however, their sample sizes were quite low, and the tested dogs were not reported to be balanced within the breeds for potential influencing factors.

Five breeds appeared to be slower compared to beagles in the NDT: hounds, golden retrievers, cocker spaniels, border collies and Labradors. This could be due to either slower decision making, or more false negative indications (the dog first missed the baited pot and then returned to it). Although it is difficult to compare, these results somewhat contradict those of previous studies not finding a difference in the drug detection speed of German shepherd dogs, cocker spaniels and Labradors^3,65^ and reporting terriers to be slower than German shepherd dogs^3^. In this study, cocker spaniels, but not terriers, seemed slower than German shepherd dogs. It is important to note that all dogs in the aforementioned studies were trained detection dogs, while we tested companion dogs, which might explain some of the differences between the findings of these studies and our results. Here, hounds were the slowest, and even though they may have a very good sense of smell^66^, they are less suitable for detection work due to their working speed. This emphasises the importance of olfactory efficiency apart from olfactory success, as it is crucial during operative work to consider the length of time a location must be closed/vacated.

Apart from the breed, we examined other factors that were expected to affect olfactory performance. To control for their already known effects on olfactory performance in the NDT, age and test location were included in the models of the present study but they had been discussed in detail earlier in Salamon et al.^45^. Briefly, 2–3-year-old dogs surpassed other age groups in reaching the TL, and dogs performed better indoors compared to outdoors^45^. We did not include sex and neutering status in the models here, as these did not affect olfactory performance in the NDT^45^.

Although both Activity/Excitability and Responsiveness to training personality traits were previously reported to be preferred characteristics of detection dogs^23,24^, we did not find an effect of Activity/Excitability. Höglin et al.^67^ showed that herding breeds had higher Activity/Excitability scores than hunting breeds and ancient breeds, while Niazy et al.^68^ found a great variation in the Activity/Excitability scores of many breeds, thus it may be possible that any effect regarding Activity/Excitability is somewhat masked by the effect of breed. Our results on Responsiveness to training scores, however, were in line with previous studies showing a positive influence on performance (e.g^34^). This reflects that better controllability, trainability and ability to concentrate, which were linked to this personality trait, contribute to better performance even in a simple but unfamiliar task like the NDT.

In contrast to previous results in different tasks (e.g^36,37,39^), ADHD total score did not affect olfactory performance. This might be explained by the fact that the NDT is based on natural motivation, thus it does not require specific attention. Impulsivity, however, may have affected dogs’ signalling style (not assessed here) rather than their performance. Although in a touchscreen test, Bunford et al.^36^ showed that a higher hyperactivity/impulsivity score in dogs was associated with a higher percentage of errors, the go/no-go tasks required considerable self-restraint from the dog, which was less important in our test.

Interestingly, the success in the olfactory test was not affected by the dogs’ training level reported by the owners. Even dogs that received olfactory type of training as family companions or detection dogs, did not perform better. This is in contrast to previous studies in other tasks where training increased performance (e.g^41,69^), which may be due to the more demanding task situations or the different assessment of training level/experience and its possible interactions with some other factors in those studies. Surprisingly, dogs with higher training levels were even slower to complete the last successful level, suggesting that prior training experience not only does not increase the dogs’ success, but rather may somehow interfere with the completion of the task. This finding may be explained by the fact that more trained dogs might have tried to solve the task by linking it to other familiar training situations, or dogs that were trained to signal in a task situation were a little more uncertain when they had to spontaneously signal an untrained decoy odour.

A recurring problem in most choice tests is that the motivation level of the subjects can significantly affect success, but it is quite challenging to measure. While in detection dogs both the selection and the training are based on dogs’ ‘toy-drive’, as they are mostly motivated and rewarded by a ball^23^, studies using family dogs may generally opt to use food as a natural motivator (e.g^14,45^). The important role of motivation in olfactory tasks is reflected by the results of Hall et al.^44^, who reported that in an anise extract detection task greyhounds (in contrast to pugs and German shepherd dogs) failed to meet the motivation criterion and could not be tested. In our study, there was no effect of the owners’ reported rewarding style, that is, whether the owner exclusively motivates the dog with food in task situations. This suggests that the differences in the general food-motivation of the subjects might not play a significant role in the success, because dogs rewarded only with food in everyday life were not likely to be more eager to seek and/or persist in the NDT. Since we used a salmon-based bait in the NDT, olfactory preference, whether the subjects liked fish smell or not, may have also affected the dogs’ motivation^70^.

This study shows that the NDT is indeed a feasible tool to test olfactory performance of dogs. It allows the testing of untrained, not pre-selected subjects of very different breeds, as well as the effect of several potentially influencing factors on the same sample, with which generalisable conclusions can be drawn about olfactory performance for the entire species. Though performance in the NDT seems to be affected by numerous genetic (e.g., breed) and environmental (e.g., training) factors and their potential interactions (e.g., Responsiveness to training), it may help select young or inexperienced dogs for detection work. Of note, apart from the ability to “hunt” the primary reinforcer, other aspects such as arousal level, environmental soundness (e.g., basophobia, dog/human fear/aggression), motivation to work for long periods of time in the absence of any visual cues of the target, propensity to learn a passive or active trained final response, and behave appropriately at the odour source, are also important factors into what makes an effective detection dog. Considering the complex nature of the task of professional detection dogs, the NDT may provide more information about inexperienced dogs’ olfactory ability than on their potential effectiveness in detection work. Obviously, even with about 500 dogs tested, this study did not allow for all possible influencing factors to be taken into account, yet this is the most comprehensive study so far on family dogs’ olfactory performance.

## Electronic supplementary material

Below is the link to the electronic supplementary material.

Supplementary Material 1

Supplementary Material 2

## Data Availability

All data are available in the Supplementary Data.
