# Supplementary material for: Breed differences in olfactory performance of dogs
Source: Sci Rep. 2025 Jan 21;15:2675. doi: 10.1038/s41598-025-87136-y (PMC11751464; doi:10.1038/s41598-025-87136-y)
Supplement: Supplementary file 2 — Supplementary Material 2 [file 41598_2025_87136_MOESM2_ESM.docx]

**Supplementary information to:**

**Breed differences in olfactory performance of dogs**

Attila Salamon^1,2,3,4*^, Ádám Miklósi^1,3^, László Róbert Zsiros^2,3^, Tímea Kovács^3,5^, Enikő Kubinyi^2,3,6^, Attila Andics^2,7^ and Márta Gácsi^1,2,3,4^

^1^HUN-REN-ELTE Comparative Ethology Research Group, Budapest, Hungary

^2^NAP Canine Brain Research Group, ELTE Eötvös Loránd University, Budapest, Hungary

^3^Department of Ethology, ELTE Eötvös Loránd University, Budapest, Hungary

^4^Hungarian Ethology Foundation, Göd, Hungary

^5^Doctoral School of Biology, Institute of Biology, ELTE Eötvös Loránd University, Budapest, Hungary

^6^MTA-ELTE Lendület “Momentum” Companion Animal Research Group, Budapest, Hungary

^7^Neuroethology of Communication Lab, Department of Ethology, Eötvös Loránd University, Budapest, Hungary

^*^Corresponding author e-mails: dr.attila.salamon@gmail.com, salamon.attila@ttk.elte.hu

**Supplementary Table S1.** Grouping of the tested breeds included in the breed group analysis (N = 484); whether they were selected for direct visual cooperation, olfaction or both.

|  | Selected for olfaction | Not selected for olfaction |
| --- | --- | --- |
| Selected for direct visual cooperation | Cocker spaniel (N = 58)  German short-haired pointer (N = 16)  German wirehaired pointer (N = 5)  Golden retriever (N = 41)  Hungarian short-haired vizsla (N = 37)  Hungarian wirehaired vizsla (N = 7)  Labrador retriever (N = 57)  Small Münsterlander (N = 1)  Weimaraner (N = 5) | Belgian Malinois (N = 29)  Border collie (N = 60)  German shepherd (N = 37) Groenendael (N = 5) Pumi (N = 2)  Tervuren (N = 4)  German × Belgian shepherd crosses (N = 3) |
| Not selected for direct visual cooperation | Basset hound (N = 14)  Bavarian hound (N = 6)  Bedlington terrier (N = 1)  Beagle (N = 43)  Bull terrier (N = 1)  Cairn terrier (N = 1)  Dachshund (N = 1)  English bloodhound (N = 7) Fox terrier (N = 1)  Hanover hound (N = 3)  Ibizan hound (N = 1)  Jack Russell terrier (N = 27)  Parson Russell terrier (N = 9)  Petit Basset Griffon Vendéen (N = 1)  Scottish terrier (N = 1) | – |

**Supplementary Table S2.** Grouping of the tested 10 breeds based on their original function included in the breed analysis (N = 439); whether they were selected for direct visual cooperation, olfaction or both. The term ‘breed’ was used rather loosely for the breed analysis, as some closely related variants were merged to maximise the number of dogs in a breed.

|  | Selected for olfaction | Not selected for olfaction |
| --- | --- | --- |
| Selected for direct visual cooperation | Cocker spaniel (N = 53)  Golden retriever (N = 38)  Labrador retriever (N = 53)  Vizslas (including short- and wirehaired Hungarian, short- and wirehaired German pointer, and Weimaraner) (N = 69) | Belgian shepherds (including Malinois, Tervuren and Groenendael) (N = 35) Border collie (N = 60)  German shepherd (N = 35) |
| Not selected for direct visual cooperation | Beagle (N = 41)  Hounds (including English, Bavarian, Hanover, Basset, and Petit Basset Griffon Vendéen) (N = 25)  Russell terriers (including Jack and Parson) (N = 30) | – |

**Supplementary Table S3.** Measured variables related to the performance and the calculation of the Success Score (SUS). The subjects had a maximum of 12 trials on each level and they passed a level, if they had at least 3 correct choices from the last 4 trials.

| Passed level | Number of trials (Level 2) | Number of trials (Level 3) | Top Level | Success Score |
| --- | --- | --- | --- | --- |
| 1 | – | – | 0 | 1 |
| 2 | 5–12 | – | 0 | 1 |
| 2 | 3–4 | – | 0 | 2 |
| 3 | 5–12 | 5–12 | 1 | 2 |
| 3 | 3–4 | 5–12 | 1 | 3 |
| 3 | 5–12 | 3–4 | 1 | 3 |
| 3 | 3–4 | 3–4 | 1 | 4 |

**Supplementary Table S4.** Odds ratios and the lower and upper 95% of their Credibility Intervals (CI) for the breeds, personality (DPQ), ADHD total, training level, reward and age examined in relation to the Top Level, Success Score and Successful Level Latency variables. The border collie was the reference category for the Top Level and the Success Score, the beagle was the reference category for the Successful Level Latency. Their odds ratios are marked as “Ref”. The effect of a factor can be considered significant if 1 does not fall between the L-95% CI and U-95% CI values. The uncertainty of the effect of a factor increases with the width of the CI range, and the closer the centre of the range is to 1. Hounds include basset and bloodhounds.

|  | Top Level | | | Success Score | | | Successful Level Latency | | |
| --- | --- | --- | --- | --- | --- | --- | --- | --- | --- |
|  | Odds ratio | L-95% CI | U-95% CI | Odds ratio | L-95% CI | U-95% CI | Odds ratio | L-95% CI | U-95% CI |
| Intercept 1 | 0.13 | 0.01 | 1.08 | 0.89 | 0.15 | 5.09 | 19.51 | 10.17 | 37.93 |
| Intercept 2 | – | – | – | 5.8 | 0.97 | 32.91 | – | – | – |
| Intercept 3 | – | – | – | 12.9 | 2.13 | 74.37 | – | – | – |
| Beagle | 0.57 | 0.24 | 1.36 | 0.84 | 0.41 | 1.81 | Ref | – | – |
| Hounds | 0.57 | 0.21 | 1.56 | 0.41 | 0.16 | 0.97 | 1.53 | 1.23 | 1.91 |
| Russell terriers | 1.01 | 0.4 | 2.62 | 1.24 | 0.54 | 2.92 | 1.22 | 0.99 | 1.52 |
| Cocker spaniel | 1.1 | 0.5 | 2.4 | 0.68 | 0.33 | 1.36 | 1.29 | 1.08 | 1.56 |
| Vizslas | 0.5 | 0.24 | 1.05 | 0.56 | 0.3 | 1.05 | 1.13 | 0.95 | 1.35 |
| Golden retriever | 0.3 | 0.12 | 0.71 | 0.26 | 0.11 | 0.56 | 1.38 | 1.13 | 1.69 |
| Labrador | 0.78 | 0.35 | 1.71 | 0.74 | 0.38 | 1.47 | 1.22 | 1.03 | 1.47 |
| Belgian shepherd | 0.88 | 0.36 | 2.18 | 0.71 | 0.33 | 1.55 | 1.14 | 0.93 | 1.41 |
| German shepherd | 0.76 | 0.3 | 1.83 | 0.64 | 0.28 | 1.43 | 1.12 | 0.91 | 1.37 |
| Border collie | Ref | – | – | Ref | – | – | 1.31 | 1.08 | 1.57 |
| DPQ Activity | 1.29 | 0.93 | 1.86 | 1.21 | 0.9 | 1.63 | 0.97 | 0.91 | 1.05 |
| DPQ Responsiveness to training | 1.33 | 1.05 | 1.69 | 1.25 | 1.03 | 1.53 | 0.98 | 0.94 | 1.03 |
| ADHD total | 0.99 | 0.96 | 1.03 | 1 | 0.97 | 1.03 | 1 | 1 | 1.01 |
| Training level | 1.07 | 0.88 | 1.29 | 1.05 | 0.9 | 1.24 | 1.05 | 1.01 | 1.09 |
| Rewarding style | 0.76 | 0.49 | 1.21 | 0.85 | 0.57 | 1.27 | 1 | 0.91 | 1.1 |
| Age | 0.93 | 0.87 | 0.98 | 0.95 | 0.9 | 1 | 1.01 | 0.99 | 1.02 |

**
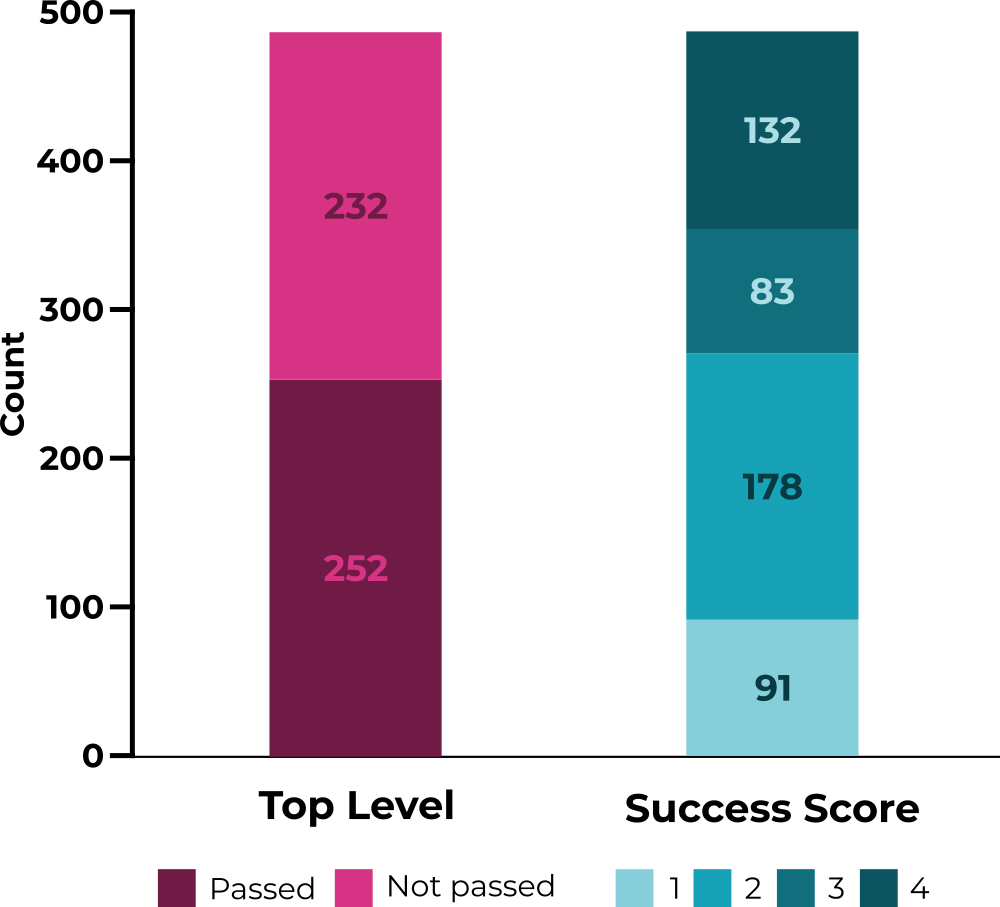
**

**Supplementary Figure S1.** Descriptive results/distribution of the two metrics, Top Level and Success Score, used to characterise the performance of dogs (N = 484) in the olfactory test.


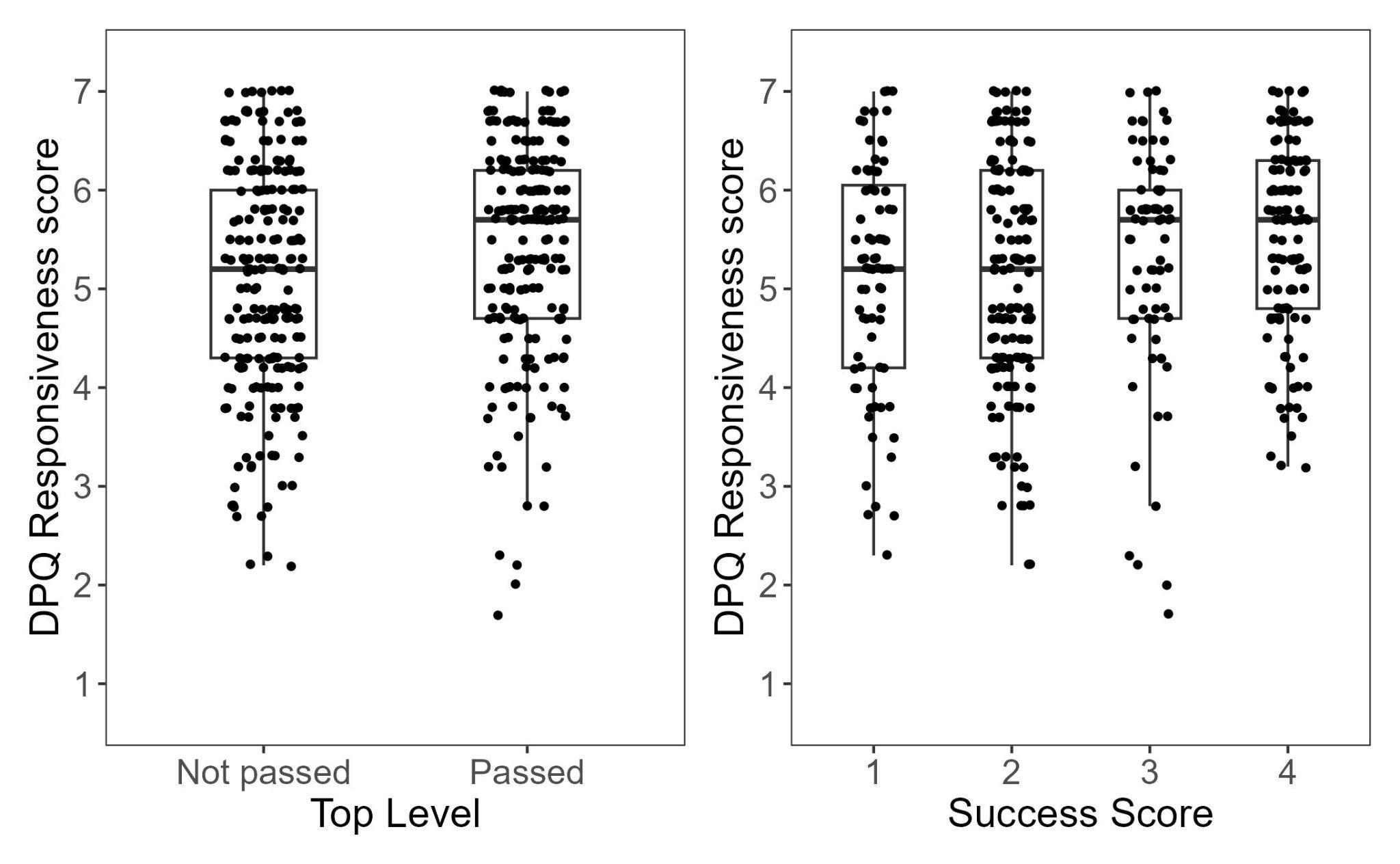


**Supplementary Figure S2.** Dogs’ Responsiveness to training scores in relation to the Top Level and Success Score metrics.
